# Immunotherapies Targeting CD123 and CD303: A New Frontier in Treating Blastic Plasmacytoid Dendritic Cell Neoplasm

**DOI:** 10.3390/ijms26062732

**Published:** 2025-03-18

**Authors:** Domenico Galati, Serena Zanotta, Fabrizia Florio, Sara Mele, Rosaria De Filippi, Antonio Pinto

**Affiliations:** 1 Hematology-Oncology and Stem-Cell Transplantation Unit, Department of Onco-Hematology and Innovative Diagnostics, Istituto Nazionale Tumori—IRCCS—Fondazione G. Pascale, 80131 Napoli, Italy; s.zanotta@istitutotumori.na.it (S.Z.); fabrizia.florio@istitutotumori.na.it (F.F.); s.mele@istitutotumori.na.it (S.M.); a.pinto@istitutotumori.na.it (A.P.); 2 Department of Clinical Medicine and Surgery, Università degli Studi di Napoli Federico II, 80131 Napoli, Italy; rdefilip@unina.it

**Keywords:** BPDCN, pDC, CD123, CD303, cancer therapy, immunotherapy

## Abstract

Blastic plasmacytoid dendritic cell neoplasm (BPDCN) is a rare and aggressive hematologic malignancy characterized by the overexpression of CD123 and CD303 surface antigens. These molecular markers play a crucial role in diagnosing diseases and developing targeted therapies. Traditional treatment options for BPDCN have demonstrated limited effectiveness, highlighting the need for new and innovative therapeutic strategies. Recent advances in immunotherapy, particularly therapeutic monoclonal antibodies, bispecific T-cell engagers, and CAR T-cell therapy, have provided promising alternatives. Tagraxofusp, the first FDA-approved CD123-targeted therapy, has significantly improved patient outcomes. Additionally, emerging CD303-targeting strategies offer the potential for further advancements. Despite these breakthroughs, challenges such as treatment resistance and toxicity remain. This review explores the latest developments in BPDCN treatment, emphasizing the potential of CD123 and CD303 as targets for precision medicine interventions. The ongoing evolution of targeted immunotherapies holds promise for improving patient survival and redefining treatment paradigms in hematologic malignancies.

## 1. BPDCN

### 1.1. Introduction

**Figure 1:**
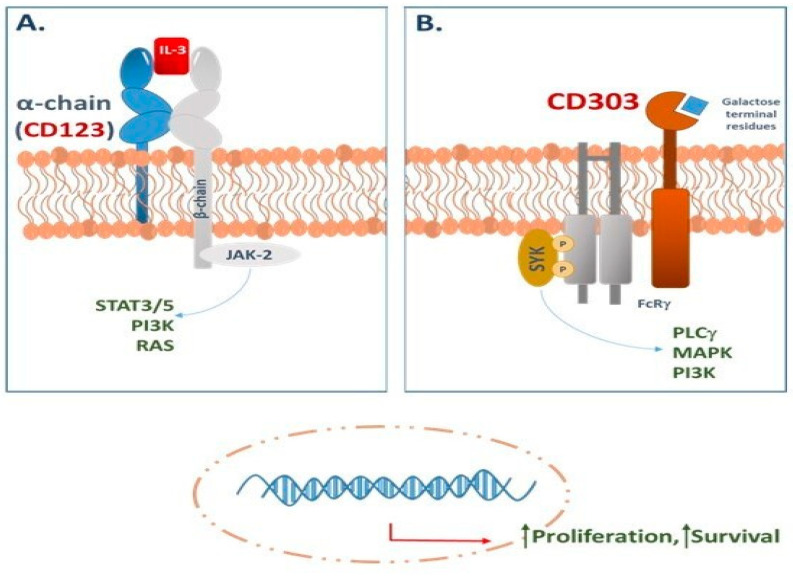
(**A**) CD123, as the alpha subunit of the IL-3 receptor, guides hematopoietic cell maturation. When bound to IL-3, it activates the beta chain, creating a dynamic IL-3 receptor heterodimer. This receptor orchestrates crucial signaling pathways for anti-apoptotic and cell-proliferative signals. (**B**) CD303 (BDCA-2) is a type II C-type lectin receptor on pDCs. It signals via the FcRγ chain, which contains ITAM motifs. Ligand binding triggers ITAM phosphorylation by Src kinases (e.g., Lck, Fyn), activating Syk and downstream pathways that regulate antigen uptake, endocytosis, and type I interferon production.

Blastic plasmacytoid dendritic cell neoplasm (BPDCN) is a rare yet highly aggressive hematologic malignancy characterized by distinct clinical and biological features. Accounting for under 1% of all hematologic malignancies [1,2], this disease predominantly arises in older individuals, typically presenting in the sixth decade of life. Epidemiological data further reveal a higher incidence among males and a slight predominance in Caucasian populations. This neoplasm originates from the precursors of plasmacytoid dendritic cells (pDCs) [2,3,4], which are a specialized subset of immune cells known for their role in the immune response, particularly through the secretion of interferon-alpha (IFN-α) during viral infections and a Th2 polarization following Interleukin-3 (IL-3) induction [1,2,5,6,7,8,9]. One of the most striking characteristics of BPDCN is the pronounced overexpression of CD123, also known as interleukin-3 receptor alpha (IL-3Rα) [9] (Figure 1A). This marker has become pivotal not only for the diagnosis of BPDCN but also for the development of targeted therapeutic strategies aimed at this malignancy. Historically, the aggressive nature of BPDCN and the limited effectiveness of available treatment options have resulted in poor prognoses for patients affected by this condition [10].

Recent advancements in our understanding of the molecular and cellular mechanisms underlying BPDCN have led to significant progress in therapeutic strategies. In particular, the identification of critical biomarkers such as CD123 and CD303 (BDCA-2) has opened new avenues for novel, targeted treatment approaches [11,12,13,14]. These novel therapies are designed to selectively target malignant cells, thus minimizing damage to healthy tissues and leading to a transformative shift in the management of BPDCN.

This review aims to explore the current advancements in BPDCN therapies, with a particular focus on the precision targeting of CD123 and CD303. Additionally, it will discuss the broader implications of these developments for patient care and the future direction of oncology research, contributing to the ongoing evolution of treatment paradigms in hematologic malignancies.

### 1.2. The Role of Plasmacytoid Dendritic Cells (pDCs) in Immunity

A comprehensive understanding of the pathophysiology of BPDCN requires examining the normal functions of pDCs within the immune system. pDCs are the subtypes CD1C+ CD11C, which express CD123 and CD303. They play a crucial role as sentinels during immune responses, particularly in detecting viral infections and producing significant quantities of type I interferons, including interferon-alpha (IFN-α). Under normal circumstances, these cells are predominantly found in lymphoid tissues, bone marrow, and peripheral blood [15,16,17].

In BPDCN, malignant changes occur in the precursors of pDCs, leading to the clonal expansion of atypical cells. These neoplastic cells are characterized by distinctive surface markers, with CD123 overexpression being particularly noteworthy [2,3,4]. Such abnormal expression serves as a defining characteristic of BPDCN, facilitating not only its identification but also the formulation of targeted treatment strategies directed at these malignant cells.

### 1.3. Comprehensive Diagnostic, Immunophenotypic, and Molecular Perspectives in BPDCN

**Figure 2:**
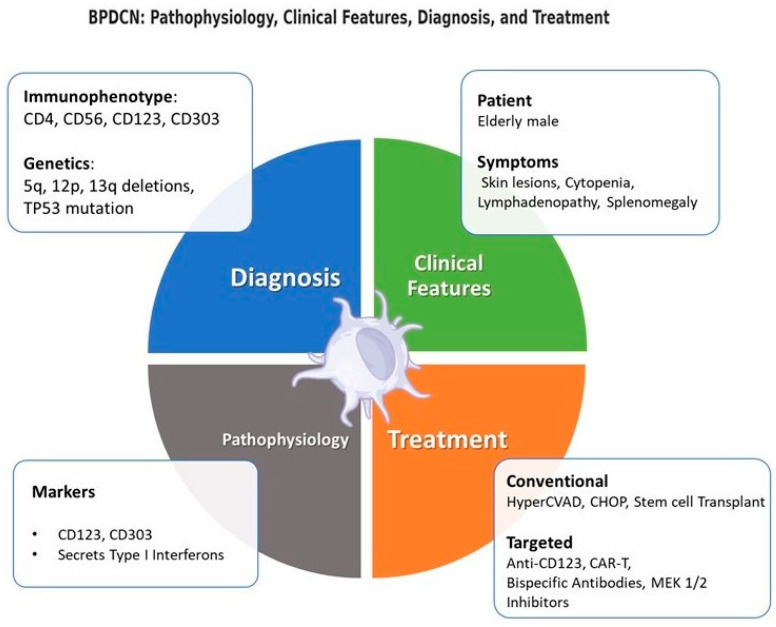
This figure provides an overview of Blastic Plasmacytoid Dendritic Cell Neoplasm (BPDCN), covering its pathophysiology (grey section), clinical features (light green section), diagnosis (blue section), and treatment options (orange section).

The accurate diagnosis of BPDCN necessitates a multidisciplinary strategy that integrates clinical, immunophenotypic, and molecular data to account for its biological complexity and phenotypic variability. Multiparametric flow cytometry (FC) is pivotal for detecting aberrant antigen co-expression—particularly CD4, CD56, CD123 (IL-3Rα), and CD303 (BDCA-2)—while excluding lineage-specific markers (Lin-). Compared to immunohistochemistry (IHC), FC provides superior sensitivity by simultaneously evaluating multiple antigens, a critical advantage for distinguishing BPDCN from immunophenotypic mimics such as NK/T-cell malignancies, cutaneous T-cell lymphoma, or CD56+ myeloid neoplasms [8,18,19,20] (Figure 2).

Recent refinements in classification further categorize BPDCN into three maturation-based subgroups: Group 1 (CD34+ immature blasts), Group 2 (CD117+ intermediate differentiation), and Group 3 (CD34−/CD117− mature phenotype). Each subgroup is associated with distinct clinical trajectories and prognostic outcomes [6].

From a genomic standpoint, BPDCN is marked by recurrent chromosomal abnormalities (e.g., del(5q), 12p/13q rearrangements, and monosomy 9), as well as frequent mutations in epigenetic regulators such as TET2, ASXL1, and DNMT3A. Additional alterations in TP53 and anomalies on 15q further underscore its genetic heterogeneity. Next-generation sequencing (NGS) has significantly enhanced the identification of these molecular drivers, refining prognostic stratification and uncovering potential therapeutic targets [20,21,22,23] (Figure 2).

### 1.4. CD123 in BPDCN: An Emerging Therapeutic Target

However, CD123 is not just a diagnostic marker: as the alpha subunit of the interleukin-3 receptor (IL-3Ra), it regulates cell survival and proliferation by activating signaling pathways such as JAK/STAT, Ras/MAPK, and PI3K [24,25,26] (Figure 1A). Its selective overexpression in BPDCN, coupled with low expression in normal stem cells, makes it an ideal therapeutic target. Agents like Tagraxofusp (an anti-CD123 antibody-drug conjugate) exploit this vulnerability, while emerging therapies (e.g., CAR-T cells, JAK/STAT inhibitors) hold promise for improving clinical outcomes [24,25,26,27].

## 2. CD123-Targeted Therapies

### 2.1. Monoclonal Antibody-Based Therapies Targeting CD123 in Blastic Plasmacytoid Dendritic Cell Neoplasm (BPDCN)

**Figure 3:**
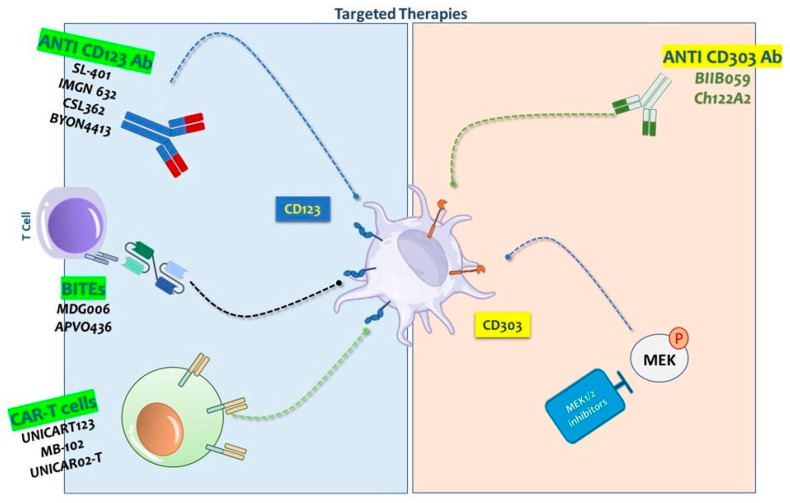
The figure depicts targeted therapies for BPDCN, focusing on CD123 and CD303 as key antigens. Anti-CD123 antibodies (SL401,IMGN632, CSL362, and BYON4413) directly target CD123 to induce cytotoxicity. Anti-CD303 antibodies (BIIB059, Ch122A2) aim at CD303-expressing cells to enhance immune clearance. BiTEs (Bispecific T-cell Engagers) (MDG006, APV0436) link T cells to BPDCN cells, promoting selective killing. CAR-T cells (UNICART123, MB-102, and UNICARo2-T) are engineered to recognize CD123 and destroy BPDCN cells. MEK inhibitors block MEK/ERK signaling, disrupting BPDCN survival and proliferation.

The increased expression of CD123 in BPDCN cells has paved the way for the development of targeted therapies, such as monoclonal antibodies and immunotoxins that specifically target this receptor [24,25,26,27]. These innovative approaches are becoming increasingly important, particularly given the high relapse rates and unsatisfactory survival outcomes linked to conventional chemotherapy (Figure 1). By concentrating efforts on CD123, there is potential for enhanced disease management and the development of more effective, less toxic treatment options for BPDCN. This marks a significant advancement in the therapeutic landscape for this aggressive form of cancer (Figure 3).

### 2.2. Tagraxofusp (SL-401)

Tagraxofusp, also known as SL-401, is the first treatment approved by the FDA specifically for BPDCN, marking a significant advancement in the fight against this challenging malignancy. This therapeutic agent is a recombinant fusion protein that merges interleukin-3 (IL-3) with a truncated form of diphtheria toxin. It harnesses the specificity of the IL-3 component to selectively target CD123, a receptor excessively expressed on BPDCN cells. This strategic configuration enables the precise delivery of the diphtheria toxin into the BPDCN cells, disrupting protein synthesis and triggering cell death through cytotoxic mechanisms [28]. It was originally used for patients with myelodysplastic syndromes (MDS) and Acute Myeloid Leukemia (AML), including a subset with BPDCN [29]. DT-IL3 was further investigated in a pilot study conducted by Frankel et al. [30]. This study specifically focused on patients with BPDCN, resulting in the agent being renamed SL-401 (Tagraxofusp, Stemline Therapeutics, New York, NY, USA) [30].

In a pivotal Phase 2 clinical trial, Tagraxofusp exhibited a remarkable overall response rate (ORR) in 70–90% of patients with previously untreated BPDCN, with the most (57% of treatment naïve) achieving a complete response (CR) [31]. The median overall survival (OS) (12–16 months) for patients treated with Tagraxofusp was significantly longer than that of those receiving conventional chemotherapy (8–12 months), representing a substantial improvement over historically poor outcomes [31]. Additionally, for patients who attained a CR, Tagraxofusp acted as a bridge to potentially curative allogeneic hematopoietic stem cell transplantation (allo-HSCT), thereby enhancing long-term survival prospects in certain cases. These results underscore Tagraxofusp’s important role not only as an effective frontline therapy but also as a bridging strategy for curative allo-HSCT in the management of BPDCN [31]. Tagraxofusp was generally well tolerated in clinical trials; however, it carries some risks such as hepatotoxicity (88%); thrombocytopenia (49%); capillary leak syndrome (CLS) (19%); and other manageable side effects, including hypoalbuminemia [31,32].

While Tagraxofusp represents a significant advancement in the treatment of BPDCN, some patients may develop resistance to the drug, resulting in a recurrence of the disease after an initial effective response. Emerging evidence suggests that resistance mechanisms in malignant cells are not primarily attributed to CD123 downregulation. Instead, they exploit DNA methylation to suppress genes required for diphthamide biosynthesis, thereby neutralizing the cytotoxic activity of diphtheria toxin [33,34,35]

Additionally, Tagraxofusp is not considered a curative treatment, and the prospects for long-term survival are particularly concerning for patients who are ineligible for stem cell transplantation. Ongoing studies aim to assess the long-term effectiveness of Tagraxofusp in preventing disease relapse, especially in those initially responsive patients who lack alternative treatment options. [31,32].

To address the limitations associated with Tagraxofusp therapy, researchers are actively exploring combination treatments. Current studies are investigating the potential advantages of pairing Tagraxofusp with agents such as 5-Azacytidine and Venetoclax, particularly for patients diagnosed with BPDCN, AML, and MDS (NCT03113643). This strategy aims to enhance therapeutic effectiveness while decreasing the risk of resistance development. Preliminary findings in high-risk AML demonstrate encouraging safety and activity, indicating a synergistic effect whereby Venetoclax promotes apoptosis in malignant cells. Meanwhile, Azacytidine modulates DNA methylation, helping to restore Tagraxofusp sensitivity, likely through the downregulation of the diphthamide biosynthesis pathway [36,37].

Ongoing and completed clinical trials are illustrated in Table 1.

In addition, next-generation CD123-targeted therapies, including bispecific antibodies and antibody-drug conjugates (ADCs), are being developed. These innovative strategies aim to improve upon the limitations of Tagraxofusp by enhancing therapeutic efficacy, reducing toxicity, and effectively addressing resistance mechanisms [38] (Figure 3).

### 2.3. Pivekimab Sunirine (IMGN632): A Next-Generation CD123-ADC with Emerging Clinical Efficacy in BPDCN

IMGN632 (Pivekimab Sunirine) has emerged as a transformative CD123-directed therapy, combining precision targeting with a novel cytotoxic mechanism. Structurally, this humanized IgG1 antibody is linked to FGN849, a DNA-alkylating indolinobenzodiazepine pseudodimer payload, enabling the selective eradication of CD123+ malignancies. Upon binding, the conjugate internalizes and releases FGN849, which induces lethal DNA damage while sparing normal hematopoietic progenitors—a critical safety advantage attributed to their inherently lower CD123 expression [39,40] (Figure 3).

Clinically, IMGN632 has redefined expectations for relapsed/refractory BPDCN. In the phase I/II CADENZA trial (NCT03386513), 30% of BPDCN patients (23 evaluable) achieved objective responses, including durable remissions (3–9 months) without transplantation. The composite complete remission rate reached 22%, with two patients attaining a sustained clinical CR [41]. Strikingly, no CLS—a hallmark toxicity of Tagraxofusp—was observed, underscoring its improved tolerability. Common adverse events (nausea, peripheral edema) were predominantly low-grade, with no grade ≥3 events recurring in > 1 patient.

Preclinically, IMGN632 demonstrates robust activity against BPDCN models, including complete regression in patient-derived xenografts and potent cytotoxicity in the CAL1 cell line [39,40]. These findings, coupled with its FDA Breakthrough Therapy Designation (2020), position IMGN632 as a cornerstone therapy for CD123+ malignancies, particularly in elderly or transplant ineligible BPDCN populations. Its unique payload mechanism and favorable safety profile further support exploration in combinatorial regimens, offering a paradigm shift in precision oncology.

In this regard, the current clinical evaluation of IMGN632 includes a Phase 1b/2 trial (NCT04086264) assessing its safety and efficacy in combination with azacitidine, venetoclax, or dual therapy for acute myeloid leukemia AML. The study’s findings, particularly regarding tolerability and therapeutic synergy, may inform the future exploration of IMGN632-based regimens in BPDCN.

Ongoing and completed clinical trials are illustrated in Table 1.

### 2.4. Other CD123-Targeted Monoclonal Antibodies Therapies

New therapeutic approaches have led to the development of monoclonal antibodies aimed at CD123. Among these, BYON4413 is a novel ADC targeting CD123, designed with a humanized IgG1 antibody that delivers a potent duocarmycin payload (Figure 3). Upon internalization, the cytotoxic agent induces DNA alkylation, leading to replication stress and cell death. Preclinical studies demonstrate its ability to selectively eliminate CD123-positive AML cell lines and patient-derived blasts while sparing CD123-negative cells. Notably, BYON4413 shows greater potency against AML blasts compared to healthy CD34^+^ hematopoietic stem/progenitor cells, highlighting its tumor-specific activity. In vivo studies using xenograft models reveal significant tumor reduction, and non-human primate data suggest a favorable toxicity profile, supporting its potential for combination therapies. A first-in-human dose-escalation trial for AML and high-risk MDS (NCT06359002) began in June 2024. With its strong preclinical efficacy and selectivity, BYON4413 emerged as a promising targeted therapy for CD123+ malignancies, including BPDCN [42,43].

CSL362 (talacotuzumab) stands out as a humanized monoclonal antibody against CD123, designed to induce antibody-dependent cell-mediated cytotoxicity (ADCC). Preclinical trials have confirmed CSL362’s ability to eliminate BPDCN cells through the activation of natural killer cells. Despite the initially promising results in clinical trials, issues such as off-target toxicity and immunogenicity persist, emphasizing need for additional studies [44,45,46] (Figure 3).

### 2.5. Bispecific T Cell Engagers

The advent of bispecific T-cell engagers (BiTEs) marks a significant advancement in the immunotherapy of CD123-expressing hematologic malignancies, particularly BPDCN and other difficult-to-treat cancers. These BiTEs are specifically designed to enhance the selective recruitment and activation of T-cells by facilitating binding interactions between CD3 receptors on T-cells and CD123 antigens on cancerous cells. This mechanism effectively positions T-cells in direct proximity to malignant cells, thereby promoting targeted cytotoxic responses (Figure 2). Currently, numerous clinical trials are underway to explore the therapeutic potential of CD123-targeted BiTEs, focusing on their safety, tolerability, and anti-tumor effectiveness [46,47,48] (Figure 3).

One notable bispecific T-cell engager, Flotetuzumab (also known as MDG006), employs Dual-Affinity Re-Targeting (DART) technology to activate CD3+ T-cells against acute myeloid leukemia (AML) cells. Flotetuzumab has demonstrated significant anti-leukemic effects in both preclinical models and clinical trials [46,47,48]. In a Phase I/II clinical trial involving patients with relapsed or refractory AML, Flotetuzumab achieved a complete response (CR) or a complete response with partial hematologic recovery (CR/CRh) rate of 26.7%, with a median overall survival (OS) of 10.2 months [47]. These findings highlight the therapeutic promise of Flotetuzumab, especially in high-risk subpopulations of AML [46,47,48]. Ongoing research, including a comprehensive basket trial (NCT04681105), is currently evaluating the efficacy of Flotetuzumab across a range of CD123- expressing malignancies (Figure 3).

APVO436 represents a significant advancement in this category as a bispecific antibody designed to target both CD123 and CD3. Preclinical evaluations reveal that APVO436 facilitates more intense T-cell activation and proliferation, along with superior depletion of CD123+ cells, compared to Flotetuzumab. Crucially, APVO436 displays a controlled cytokine release profile, which could lessen the frequency of immune-related side effects. In experiments involving subcutaneous tumor models, APVO436 significantly curtailed tumor growth and was associated with notable T-cell infiltration and activation at the tumor site, especially after the intravenous introduction of human T cells. These preliminary outcomes advocate for the further clinical exploration of APVO436 as a promising treatment option for AML, BPDCN, and other hematologic malignancies [49,50,51] (Figure 2).

The robust efficacy and its favorable safety profile suggest that APVO436 could become a foundational element in the therapeutic regime for specific hemopoietic cancers. Upcoming clinical trials are essential to corroborate these initial results and could eventually position APVO436 as a pivotal agent in the management of lympho-hemopoietic malignancies (Figure 3).

Ongoing and completed clinical trials are illustrated in Table 1.

### 2.6. Advances in CD123-Targeted Chimeric Antigen Receptor (CAR) T-Cell Therapy for BPDCN and Hematologic Malignancies

CAR T-cell therapy has emerged as a transformative strategy in cancer immunotherapy, which can be successfully applied to CD123-positive cancers such as BPDCN. This technique modifies a patient’s T cells to carry CARs that specifically target CD123, thus allowing for the precise detection and destruction of BPDCN cells. Initial studies have shown that CAR T cells directed at CD123 can robustly combat leukemia, successfully eradicating BPDCN cells in laboratory and animal studies [25]. In particular, T cells from BPDCN patients engineered with CD28/4-1BB CD123 CARs have displayed significant cytotoxic abilities in vitro against BPDCN blasts, with further studies confirming their efficacy in reducing BPDCN load while limiting collateral damage to healthy tissues [52]. A novel advancement in this area is the generation of TCRαβ-negative allogeneic CAR T cells, often termed “universal” CAR T cells. These are designed to overcome some of the limitations of autologous CAR T-cell therapies. One example, UCART123, employs T cells from healthy donors that have been engineered to target CD123 using the TALEN technology for genetic modification. UCART123 has shown considerable potential in treating relapsed or refractory AML and BPDCN. The approach may result in a particular value for patients for whom the production of viable T cells for autologous therapy is difficult or compromised [53] (Figure 2). In preclinical models, UCART123 demonstrated significant activity against BPDCN using cells directly from patients, although challenges such as antigenic shift or CD123 loss could limit its effectiveness in clinical settings [53]. Clinical trials, such as NCT03203369 and NCT04109482, are currently underway to evaluate the clinical efficacy and safety of anti-CD123 CAR T cells in BPDCN, with results still pending on ClinicalTrials.gov (Figure 3). Concurrently, several other CAR-T cell constructs are being tested. Notably, MB-102 (NCT04109482 and NCT02159495) and UniCAR02-T (NCT04230265) are targeting CD123-positive hematological malignancies, including BPDCN [25,54,55] (Figure 3). UniCAR02-T, currently in a phase I trial for relapsed AML, ALL, and BPDCN, utilizes a unique approach where UniCAR T cells are activated by a specific recombinant antibody derivative targeting CD123 (TM123), which may enhance both specificity and safety [54] (Figure 3).

Ongoing and completed clinical trials are illustrated in Table 1.

CAR-T cell therapy has demonstrated remarkable potential in treating cancers like BPDCN, yet critical challenges impede its broader use. A primary issue is antigen escape, where tumors downregulate or lose the antigen (e.g., CD123 in BPDCN) targeted by CAR-T cells, enabling relapse. Equally significant is on-target off-tumor toxicity, occurring when CAR-T cells attack healthy tissues expressing the same antigen, such as CD123 on certain epithelial or hematopoietic cells. Additionally, the immunosuppressive tumor microenvironment (TME)—rich in inhibitory cells, cytokines, and checkpoint molecules—suppresses CAR-T cell activity and persistence [56,57].

Safety remains a major concern, with toxicities like CRS and neurotoxicity posing life-threatening risks. To address these limitations, researchers are advancing engineered CAR designs, including dual-targeting systems (to prevent antigen escape), logic-gated receptors (to improve specificity), and safety switches (to deactivate CAR-T cells if toxicity arises). Combination therapies with checkpoint inhibitors or cytokine modulators are also being tested to counteract the TME [56,57].

For BPDCN, CD123 remains a compelling target due to its high expression on malignant cells. Current clinical trials focus on refining CAR-T cell constructs, optimizing dosing, and identifying biomarkers to predict response or toxicity. While hurdles persist, these innovations aim to enhance efficacy, durability, and safety, positioning CD123-directed CAR-T therapy as a transformative option for BPDCN and other hematologic malignancies. (Figure 3).

## 3. CD303-Targeted Therapies

### 3.1. CD303 Is a Useful Diagnostic Marker in BPDCN

CD303, also known as BDCA-2, DLEC, or CLEC4C, is a unique type II c-type lectin receptor (CLR) found on human pDCs. Its role is to assist TLR-7/9 in the recognition of microbes and in the coordination of an inflammatory immune response (Figure 1B). Significantly, the expression levels of CD303 decrease as pDCs reach maturity, implying a higher expression in their immature state [58,59,60]. This specificity renders CD303 a compelling target for both diagnostic and therapeutic strategies in BPDCN, marking a significant advancement in the field of hematopathology [61]. Distinct from other markers such as CD123 and CD56, which manifest across a spectrum of hematologic malignancies, CD303 is predominantly expressed on pDCs, thereby providing a potent diagnostic tool for identifying malignant pDCs in BPDCN [58,59,60]. Research highlights the diagnostic value of CD303 in BPDCN, with studies showing its expression in nearly all cases examined, thus providing high sensitivity and specificity [58,59,60]. In clinical practice, immunohistochemical staining for CD303 is routinely conducted alongside other pDC markers, such as CD123 and TCL1, to corroborate a BPDCN diagnosis [62]. This strategy is particularly invaluable given the clinical similarities BPDCN shares with other malignancies [63,64].

From a therapeutic perspective, the development of anti-CD303 antibodies and antibody-drug conjugates (ADCs) is likely to greatly improve treatment outcomes for BPDCN. While these developments are still in the preliminary stages, preclinical studies have produced promising results, and clinical trials are expected [61] (Figure 3).

### 3.2. The Role of CD303 in Targeted Therapy

Beyond its diagnostic utility, CD303 (BDCA-2) has emerged as a notable therapeutic target for BPDCN. The creation of targeted therapies for BPDCN is challenging due to the rarity of the disease and a limited array of clearly defined molecular targets.

Nevertheless, CD303’s expression on malignant pDCs provides an effective means for therapeutic intervention. Monoclonal antibodies such as Ch122A2 and BIIB059, initially created for treating systemic lupus erythematosus (SLE) by suppressing the activation of pDCs and thus diminishing immune responses linked to cutaneous lupus erythematosus (CLE), have shown promising results in the preclinical treatment of BPDCN [61,65,66] (Figure 3).

These monoclonal antibodies are designed to specifically target CD303 on the surfaces of malignant pDCs, leading to cell destruction through mechanisms such as antibody-dependent cellular cytotoxicity (ADCC) and complement-dependent cytotoxicity (CDC) [67]. This method of precision targeting enhances the specificity of the treatment and reduces potential side effects. The application of ADCC and CDC is essential for effective cytotoxicity against BPDCN cells, potentially offering a more effective and less harmful alternative to traditional chemotherapy.

Additionally, there is an escalating interest in developing small-molecule inhibitors that target pathways associated with CD303. Early studies suggest that inhibiting the MEK1/2-ERK pathway, which reduces Toll-like receptor 9 (TLR9)-mediated interferon type I (IFN-I) production in pDCs, could modify the immunogenicity of malignant pDCs through pharmacological interventions [68] (Figure 2). By modulating TLR9 signaling, MEK1/2 inhibitors could decrease BPDCN cell proliferation and enhance the efficacy of immunotherapeutic approaches. The direct targeting of TLR9, including strategies like CpG(A)-STAT3 small interfering RNA (siRNA) conjugates, has demonstrated in vitro activity by inducing TLR9-dependent gene silencing and boosting immune responses in dendritic cells, including pDCs [69].

This strategy may enhance the anti-tumor activity of pDCs by improving their ability to stimulate the immune system, providing an indirect way to limit BPDCN cell growth. While the specific signaling roles of CD303 are still under investigation, it is believed to influence pDC functions, including interferon production modulation. By targeting CD303-associated pathways, small-molecule inhibitors could effectively reduce BPDCN cell activity and control tumor progression. Investigations into the role of CD303 in the biology and immune regulation of BPDCN are expected to result in novel, mechanism-based therapies tailored to the unique molecular characteristics of BPDCN (Figure 3).

## 4. Conclusions

Recent breakthroughs in understanding and treating BPDCN have revolutionized its management, providing new hope for improved patient outcomes in this aggressive malignancy. Key to these advancements has been discovering specific markers such as CD123 and CD303, which have enabled the development of targeted therapies. These therapies, including the CD123-directed Tagraxofusp and various immunotherapeutic agents, specifically target malignant cells, thereby sparing healthy tissues and demonstrating significant effectiveness in clinical settings. This marks a substantial improvement over traditional chemotherapy options. Despite recent advancements, challenges such as treatment resistance, toxicity, and the potential for relapse continue to pose significant obstacles that affect long-term remission rates for many patients.

In response, ongoing research is concentrating on innovative strategies, including combination therapies and bispecific antibodies, with the goal of achieving sustained remission and potentially curing the disease. Although CD123- and CD303-targeted therapies have significantly advanced BPDCN treatment, their heterogeneous expression in patients calls for the identification of supplementary biomarkers. Future research should focus on exploring alternative targets, such as CD56, TCL1, and recurrent genetic alterations (e.g., TET2, ASXL1, TP53), to enhance treatment specificity and efficacy, ultimately broadening the clinical applicability of precision medicine in BPDCN [23].

The management of BPDCN is expected to become more personalized by combining novel and conventional treatments to improve patient outcomes and combat resistance. The ongoing development of therapies targeting CD123 and CD303 is crucial, offering more precise and less harmful options. These advancements signal a brighter future for BPDCN patients, highlighting the potential of targeted therapies in hematologic oncology.

## Figures and Tables

**Table 1:** Ongoing and completed clinical trials.

Study ID	Therapeutic Strategy	Condition/Disease	Phase	Status
**NCT03113643**	***Tagraxofusp*** + azacitidine ± venetoclax	AML, MDS, and BPDCN	**I**	**Recruiting**
**NCT03386513**	***IMGN632***	AML, ALL, BPDCN, MPN	**I/II**	**Active, not recruiting**
**NCT04086264**	***IMGN632*** alone or + azacitidine ± venetoclax	CD123-Positive AML	**I/II**	**Active, not recruiting**
**NCT06359002**	***BYON4413***	R/R AML, and MDS	**I**	**Recruiting**
**NCT04681105**	Bispecific antibodies ***Flotetuzumab***	AML, BPDCN	**I**	**Active, not recruiting**
**NCT03203369**	***UCART123***	BPDCN	**I**	**Terminated**
**NCT04109482**	Chimeric Antigen Receptor T cells ***MB-102***	BPDCN	**I/II**	**Terminated**
**NCT02159495**	Chimeric Antigen Receptor T cells ***CD123^+^ CAR T cells***	AML, BPDCN	**I**	**Active, not recruiting**
**NCT04230265**	Chimeric Antigen Receptor T cells ***UniCAR02-T + TM123***	AML, BPDCN	**I**	**Recruiting**

## Data Availability

Not applicable.
